# Heat Shock Proteins in Medically Relevant Fungal Pathogens: From Molecular Chaperones to Virulence Factors and Therapeutic Targets

**DOI:** 10.3390/jof12060414

**Published:** 2026-06-07

**Authors:** Leonardo Padró-Villegas, Héctor M. Mora-Montes

**Affiliations:** Departamento de Biología, División de Ciencias Naturales y Exactas, Campus Guanajuato, Universidad de Guanajuato, Noria Alta s/n, col. Noria Alta, Guanajuato 36050, Gto., Mexico; l.padrovillegas@ugto.mx

**Keywords:** antifungal resistance, virulence factors, fungal infections, cell adhesion, thermotolerance, chaperones

## Abstract

Heat shock proteins (HSPs) are highly conserved molecular chaperones that play a key role in maintaining protein homeostasis and cellular survival under stress conditions. Clinically relevant human pathogenic fungi include opportunistic fungi, dimorphic fungi, dermatophytes, Mucorales, and other pathogenic groups. HSPs, including Hsp90, Hsp70, Hsp60, Hsp40, and Hsp110, are essential for the correct nascent protein folding, aggregation prevention, and degradation of misfolded polypeptides. Fungal pathogens frequently encounter environmental and host-imposed stresses, including oxidative stress, temperature fluctuations, and antifungal treatments. This review synthesizes and critically analyzes current evidence on the role of HSP families in essential processes linked to fungal virulence, including morphogenetic transitions, biofilm formation, maintenance of cell wall integrity, and interactions with host immune cells. Beyond their canonical chaperone functions, HSPs act as central mediators in pathogenic processes, such as morphogenesis transitions, biofilm formation, cell wall integrity, and interactions with host immune cells. Hsp90 stabilizes key signaling proteins involved in stress responses, morphogenesis, and antifungal resistance, while Hsp60 and Hsp70 contribute to mitochondrial function, cell wall integrity, and immune modulation. Disruption of these chaperones impairs growth, reduces virulence, and increases susceptibility to antifungal agents. The rise of antifungal resistance underscores the urgent need for new therapeutic strategies. Targeting fungal HSPs has emerged as a promising approach due to their essential roles in stress tolerance and pathogenesis. Hsp90 inhibitors, including geldanamycin derivatives and other small molecules, have demonstrated the ability to impair fungal growth, reduce virulence traits, and sensitize resistant strains to conventional antifungal drugs. Combining HSP inhibitors with existing antifungal drugs represents a potential strategy to overcome resistance and improve treatment outcomes. This review summarizes the current knowledge on HSPs in pathogenic fungi, focusing on their roles in stress adaptation, virulence, host-pathogen interaction, antifungal resistance, and their potential as targets for novel antifungal therapies.

## 1. Introduction

All organisms develop strategies to respond to environmental stress, such as abrupt changes in temperature, salinity, pressure, and exposure to radiation or oxidizing agents, among others. These can damage cellular components, such as the cell membrane, cell wall, internal organelles, and even genetic material. So, strategies to cope with stress are essential to maintain cell viability. Several genes with the ability to respond to environmental stimuli are conserved from ancestral prokaryotic organisms to higher eukaryotic species. The response to environmental stress triggers a series of intracellular signaling events that lead to the activation/deactivation of specific gene expression regulons, controlling the pre- and post-translational events during protein synthesis [1]. Thermal stress can affect normal cellular function by denaturing proteins and preventing their correct synthesis. Therefore, there is a gene family that encodes proteins known as molecular chaperones, which are highly conserved in all organisms and are responsible for protein homeostasis, regulating the correct folding of new proteins, helping to maintain their native conformation under stress conditions, preventing protein aggregation, and helping in the degradation of misfolded proteins [1,2]. Molecular chaperones are a protein family with heterogeneous molecular weights, ranging from 10 to 100 kDa. They perform their function by forming rings, arranged by several units of the same chaperone, forming oligomeric complexes containing from 6 to 50 subunits, carrying out their molecular function in the cytoplasm or some other subcellular compartment [2,3].

Since the seventies, these chaperones were described in *Escherichia coli* and are encoded by genes of the *GroE* group, among which two genes encode a 60 kDa and a 12 kDa protein, identified as GroEL and GroES, respectively. Moreover, the 70 kDa chaperone DnaK was identified, which works in collaboration with the 40 kDa co-chaperones DnaJ and GrpE that help DnaK in ATP hydrolysis and substrate specificity. The DnaK-DnaJ-GrpE complex collaborates with the 90 kDa chaperone HtpG to generate a protein folding system that is upregulated under high-temperature conditions and is essential for normal cell growth [4,5,6,7,8]. This DnaK-DnaJ-GrpE-HtpG complex is assisted by other ATPases, such as ClpA, ClpB, ClpX, and ClpY. This chaperone family was also described in the fruit fly, *Drosophila melanogaster*, where they were named heat shock proteins or HSPs, specifically for eukaryotic cells, because the main overexpression of the 70 kDa chaperone (Hsp70) was observed when the flies were subjected to heat stress [9]. This protein family was also identified in the chloroplasts of *Arabidopsis thaliana*, where they were named as chaperonins, which included the chaperone Hsp60 (GroEL) and its co-chaperone Hsp10 (GroES). Furthermore, the following were identified: Hsp70 (DnaK), involved in hyperthermal stress; Hsp90 (HtpG), related to the correct plant development; and a group of low molecular weight chaperones, such as Hsp20, upregulated under low-temperature conditions, which has been reported in some species of psychrophilic plants [10,11,12]. In humans, HSPs function as molecular chaperones, and their dysfunction has been related to some neurodegenerative diseases, like Parkinson’s and Alzheimer’s, due to the formation of protein aggregates of α-synuclein and β-amyloid plaques, respectively. They have also been associated with the development of diverse types of cancer, due to their participation in processes such as the cell cycle, apoptosis, and signaling processes [2,13,14].

This review aims to provide a comprehensive overview of the roles of HSPs in medically important pathogenic fungi, focusing on their contribution to cellular proteostasis, stress adaptation, virulence, host-pathogen interactions, and antifungal drug resistance. In addition, this review summarizes current advances in targeting fungal HSPs as potential therapeutic strategies and discusses their relevance as emerging antifungal drug targets.

## 2. Fungal Infections

The most persistent fungal infections are caused by opportunistic fungal pathogens, especially those caused by members of the *Candida* genus [15]. Causative agents are normally found to be part of the human microbiota and can proliferate and damage the host tissues when immunity is compromised. The most frequent agents of candidiasis are *Candida albicans*, *Candida auris*, *Candida glabrata*, *Candida parapsilosis*, and *Candida tropicalis.* These fungal pathogens undergo dimorphism when growing within the host’s tissues, going from yeast to hyphae [15]. Also, among the most prevalent opportunistic fungal infections are aspergillosis and cryptococcosis, which are characterized by the colonization of the respiratory system, generating a localized infection in the lungs, with atypical lesions in other tissues, like mucous membranes, skin, lymph nodes, brain, and adrenal glands [16,17]. *Aspergillus fumigatus* and *Cryptococcus neoformans* are etiological agents of aspergillosis and cryptococcosis, respectively [17,18]. Other pulmonary opportunistic infections are caused by fungi with different biological characteristics, such as the yeast fungus *Trichosporon asahii* and the filamentous fungus *Lomentospora prolificans* (formerly *Scedosporium prolificans*). Fungi can infect the respiratory system. *T. asahii* is associated with hypersensitivity pneumonitis, whereas *L. prolificans* can cause severe and often fatal pulmonary infection [19,20]. The mucoralean fungi are opportunistic pathogens responsible for different clinical manifestations of mucormycoses, including necrotizing mucocutaneous mucormycosis. These infections are invasive and commonly associated with penetrating trauma or burn injuries. In the late stages of the disease, mucormycosis presents high morbidity and mortality. *Rhizopus arrhizus*, *Rhizomucor pusillus*, and *Mucor circinelloides* are among the most common causative agents of mucormycosis [21].

The dimorphic fungi *Histoplasma capsulatum*, *Paracoccidioides brasiliensis*, and *Coccidioides immitis* are fungal pathogens that generate pulmonary infection, namely, histoplasmosis, paracoccidioidomycosis, and coccidiomycosis. These fungal infections can lead to chronic granulomatous pneumonia. *H. capsulatum* and *P. brasiliensis* are mainly present in tropical and subtropical regions in Latin America, whereas *C. immitis* is found in semi-arid regions such as the deserts of Arizona and Texas. These fungi undergo a morphological transition from the hyphal form (saprophytic phase) in the environment to yeast-like cells (pathogenic phase) in the host [18,22,23]. Another respiratory infection with symptomatology similar to tuberculosis is caused by the dimorphic fungi *Talatomyces marneffei*, which primarily affects the regions of Southeast Asia [24]. Infections occur through inhalation of environmental conidia, leading initially to pulmonary disease that can subsequently disseminate to other organs. Another dimorphic fungal infection is sporotrichosis, known as the gardener’s infection. This is acquired by sapronotic inoculation in open wounds or the zoonotic transmission by infected cats [25]. This infection is considered a cutaneous mycosis, affecting the dermal and subdermal tissues and lymph nodes. In some cases, it can affect the mucous membranes and lungs and generate a disseminated form [26]. The etiological agents are the species of the *Sporothrix* genus, including *Sporothrix schenckii*, *Sporothrix brasiliensis*, *Sporothrix globosa*, and *Sporothrix lurei* [27]. These are thermodimorphic pathogens too, which grow as yeast-like cells at temperatures over 35 °C. In the case of *S. brasiliensis*, the most frequent agent of feline sporotrichosis, it can tolerate higher temperatures, as the corporal temperature of cats is closer to 39 °C [28].

*Fonsecaea pedrosoi* is a dimorphic fungus and one of the main causative agents of chromoblastomycosis. This fungal infection affects cutaneous and subcutaneous tissues and is characterized by granulomatous and suppurative mycosis [29]. Dermatophyte fungal infections are also important to human health because these fungi can grow in non-conventional structures such as skin, nails, and hair. This ability is due to the enzymatic machinery that allows them to metabolize keratin. *Trichophyton rubrum* and *Trichophyton mentagrophytes* are among the main agents of nail mycosis, known as onychomycoses [30,31]. Species of the genus *Malassezia* include yeast cells such as *Malassezia sympodialis*, which are considered commensals of adult skin but can also generate opportunistic infections, like atopic eczema/dermatitis syndrome [32].

## 3. Canonical Function of Fungal HSPs

Like all organisms, fungi have managed to develop strategies to tolerate different types of environmental stress, including the response to thermal stress, so the presence of these chaperones in these organisms has been a fundamental part of their survival and adaptation to new environments. Therefore, these chaperones have been of great interest in understanding fungal biology.

### 3.1. The 110 kDa Heat Shock Protein

Structurally, Hsp110s are composed of two main domains, an N-terminal domain (NTD) and a C-terminal domain (CTD). In most cases, the NTD is a 45 kDa region that exerts the activity of binding to substrates, ATP, or nucleotides. In the case of the CTD region, this is a 25 kDa fragment, which is a peptide-binding domain (PBD), working in association with other proteins. The Hsp110 functions as a co-chaperone for other chaperones, such as Hsp70 and Hsp90, acting as a nucleotide exchange factor (NEF), which improves the ATPase function in these chaperones [33]. Hsp110 stimulates the Hsp70 ATPase activity, binding and releasing target proteins at the binding site, essential for the degradation of protein aggregates [33,34].

Protein homeostasis includes the correct folding of de novo-generated proteins, the degradation of misfolded proteins, and the solubilization of protein aggregates. Under thermal stress, the newly generated proteins become insolubilized and deposited, generating aggregates, which are potentially cytotoxic. Therefore, there is a network of specialized chaperones to solve this issue [33,34]. One of the players in this process is Hsp110, which is recruited after the heterodimer chaperone Hsp70 and co-chaperone Hsp40 recognize and bind to hydrophobic amino acid segments of misfolded proteins. The Hsp110-Hsp104 complex facilitates the recycling of Hsp70 by promoting its transition to the ATP-bound open conformation. The NEF of Hsp110 mediates the release of ADP and the bound substrate from Hsp70, enabling the re-engagement of the Hsp70-Hsp40 machinery at additional sites within the protein aggregate [34,35]. Together, these chaperone complexes contribute to the dissolution of the misfolded structures of the aggregated proteins, such as amyloid aggregates resulting from protein denaturation. This chaperone network culminates in the reorganization of misfolded proteins and returning them to their native state [34,35,36].

### 3.2. The 90 kDa Heat Shock Protein

The 90 kDa molecular chaperone, or Hsp90, has a highly conserved canonical function and a certain structural homology in all kingdoms of life. This chaperone forms a homodimer, in which it has three main structural domains: an NTD, a domain in the middle region (MD), and a CTD. Each domain is necessary for the full function of the chaperone, in which the NTD contains the ATP-binding pocket and ATPase activity, the latter regulated by the MD region. Finally, the CTD regulates the homodimerization of the Hsp90 subunits, and the presence of an EEVD structural domain allows the binding of the co-chaperone Hsp70. In general, CTD controls the correct protein assembly, favoring its binding to target proteins [37,38,39].

The Hsp90 homodimer has a naturally open conformation; with the binding of the nucleotide in the ATP-binding pocket, Hsp90 undergoes conformational changes that enable the recognition of client proteins. This conformation changes transiently to allow the binding of different target proteins. Once the ATP is hydrolyzed to ADP, it takes a closed conformation, and subsequently the nucleotide is uncoupled again, leaving the pocket free again for the binding of another ATP molecule [37,38,40]. It is thought that Hsp90 has a certain preference for proteins with intrinsically disordered regions in their structure, which do not acquire a stable conformation, mostly found as part of signaling pathways and transcription factors. So, this HSP is essential for the stabilization of signaling pathway elements, such as MAP kinases, that respond to cellular damage [38,41]. It is estimated that 8% of the *Saccharomyces cerevisiae* total proteins are targeted by this chaperone [38]. Additionally, Hsp90 is essential not only for the folding process of new proteins but also for the process of protein degradation mediated by the ubiquitin/proteasome system [42]. Conditional mutants or depletion of HSPs can strongly affect ubiquitin-mediated proteasomal degradation. Studies in *S. cerevisiae* have demonstrated that the loss of specific chaperones or co-chaperones reduces the stability of newly synthesized protein kinases, promoting their targeting to the ubiquitin/proteasome system rather than productive folding and maturation. Consequently, client proteins accumulate in misfolded states and are preferentially directed toward proteasomal degradation, highlighting the critical role of HSPs in maintaining protein homeostasis and quality control.

### 3.3. The 70 kDa Heat Shock Protein

The 70 kDa molecular chaperones, Hsp70/DnaK, are a large group of proteins that, unlike Hsp90, only have two structural domains: an NTD of approximately 45 kDa, which is a nucleotide-binding domain, and a CTD of approximately size of 25 kDa, which is responsible for binding to the target protein. The members of this chaperone group never function in isolation; they are functionally linked to other chaperones, including Hsp90 or co-chaperones, such as Hsp40/DnaJ [43].

Hsp70 recognizes short hydrophobic regions in stretches exposed in non-native or partially folded proteins and binds to these domains to prevent irreversible aggregation. Under thermal stress conditions, widespread protein destabilization increases the exposure of such hydrophobic motifs; therefore, the overexpression of these chaperones helps mitigate the damage caused by protein denaturation [38]. The Hsp70 canonical function involves its NTD region, which contains an adenine nucleotide-binding domain (NBD) that binds and hydrolyzes ATP, essential for the chaperone function, whereas the CTD domain, controlling protein refolding by the substrate-binding domain (SBD), is formed by a β-sheet peptide-binding pocket and an α-helical “lid” that controls substrate accessibility. When ATP is bound, Hsp70 adopts an open conformation with low substrate affinity. Due to the ATP hydrolysis, the CTD undergoes a conformational change that closes the helical “lid” over the bound polypeptide, stabilizing it in a high-affinity state [43,44]. However, the intrinsically low ATPase activity of Hsp70 makes this conformational change relatively slow on its own. Therefore, it depends on co-chaperones of the Hsp40/DnaJ family, which help stimulate ATPase activity [43,44].

In addition, Hsp70 has been identified as part of a mechanism of gene expression regulation, in which Hsp70 mediates gene coactivation by acting as an HSF (Heat Shock Factor) transcription factor, by joining the 5′-UAS region (Upstream Activating Sequence) of the laccase gene or directly interacting with mRNA complexes due to ARE domains (AU-rich elements) found in the 3′-UTR region, which leads to its degradation by removing the poly(A) tail and the 5′ cap. These mechanisms are related to glucose starvation and the absence of heat shock in *S. cerevisiae* and *C. neoformans* [45,46].

### 3.4. The 60 kDa Heat Shock Protein

Hsp60 or chaperonins are known as mitochondrial chaperones, since they are found mostly in this subcellular compartment. However, they are also found in the cytosol [18]. Structurally, they are formed as homopolypeptides, which are composed of fourteen Hsp60 monomers, spatially arranged by the formation of two rings with seven monomers each. The barrel-shaped structure generated an internal cavity that serves as the Hsp60 interaction point with the proteins to be folded. For this to happen, a conformational change must first occur by the binding of ATP to the Hsp60 binding pocket, optimized by the function of its co-chaperone Hsp10 [47,48]. Each Hsp60 subunit is organized by three domains: an equatorial domain with ATPase activity, responsible for ATP binding and hydrolysis; an intermediate domain that mediates conformational changes and inter-subunit communication; and an apical domain that interacts with the client protein and the co-chaperone Hsp10. Hsp60 operates through an ATP-driven cycle in which substrate binding, ATP-dependent cavity closure, and ATP hydrolysis promote folding and subsequent release. This encapsulation mechanism is especially important under stress conditions that destabilize mitochondrial proteins. Hsp70 and Hsp10 coordinate with Hsp60 during the formation of active complexes. Hsp70 stabilizes and delivers unfolded substrates, while Hsp10 acts as an ATP-dependent lid that seals the chamber and enables productive folding [47].

The Hsp60 canonical function has been related only to mitochondria; however, these have been found in different subcellular and extracellular compartments, developing a variety of functions for proteostasis, among which the large oligomeric rings that these form can encapsulate target proteins and determine their final destination, one of which is degradation. It has been reported that the cytosolic Hsp60 accumulation leads to proteasome inactivation by direct interaction with proteasomal proteins. The co-localization of these proteins in the cytosol may be due to a translocation process, resulting from conditions of thermal stress or mitochondrial damage, since it has been proven that the inhibition of the proteasome in eukaryotic cells leads to apoptosis [49].

### 3.5. The 40 kDa Heat Shock Protein

Hsp40/DnaJ is a protein family characterized by having the J domain, typically located at the N-terminal region, which is responsible for ATPase activity stimulation domain, as well as a nucleotide exchange factor. Hsp40 protein does not promote nucleotide exchange but instead enhances ATP hydrolysis in Hsp70. These co-chaperones are helpers of Hsp70, promoting the delivery and proper binding of target polypeptides to the peptide-binding site, and stimulating Hsp70 ATPase activity, inducing its conformational change required to stabilize its interaction with the client protein. Furthermore, Hsp40 interacts directly with exposed hydrophobic regions of the target polypeptides, facilitating substrate recognition and preventing premature aggregation. The coordinated opening and closing cycle favors protein folding and prevents their aggregation. This system protects nascent proteins from being degraded by the ubiquitin/proteasome system [42,50].

This set of Hsp70-Hsp40 chaperones has more functions, such as the internal transport of proteins to the endoplasmic reticulum and mitochondria, and the disaggregation process of denatured and misfolded proteins in the cytosol (see Section 3.3 for the mechanistic details) [51].

## 4. Function of HSPs as Virulence Factors in Pathogenic Fungi

**Figure 1:**
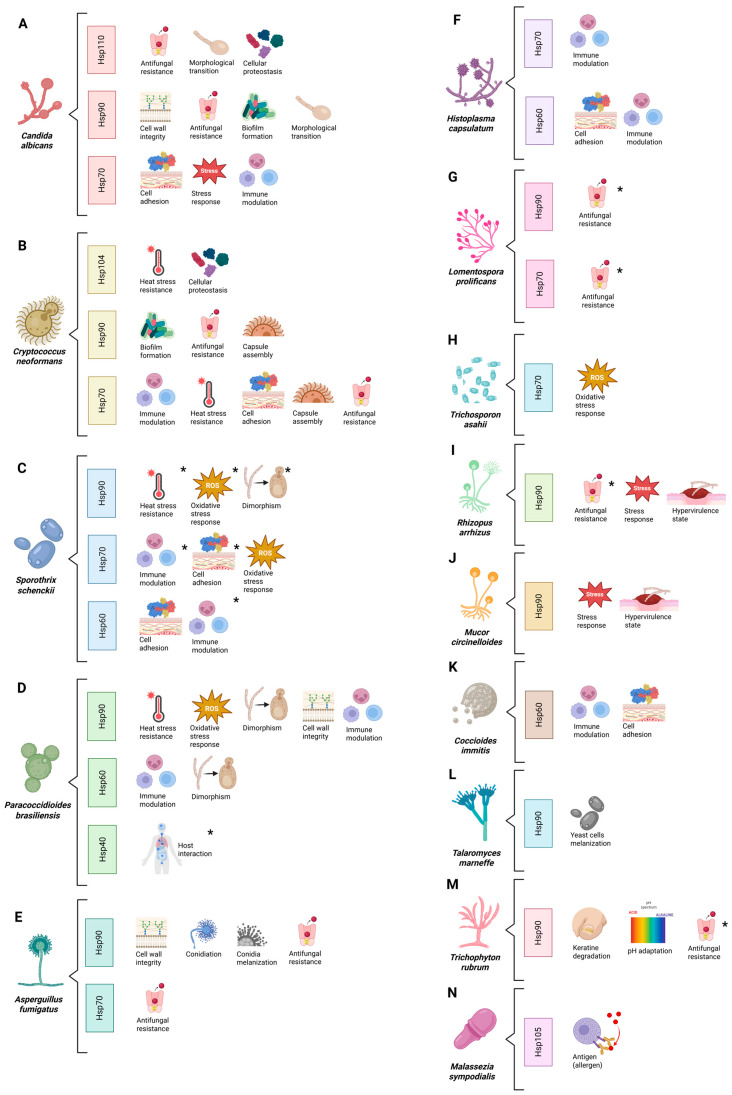
**Summary of the functions of heat shock proteins in pathogenic fungi.** Schematic representation of the non-canonical functions attributed to heat shock proteins in pathogenic fungi, including dimorphic transition, biofilm formation, antifungal resistance, immune system modulation, and cell adhesion. These functions are grouped according to the fungal species where they have been described and the heat shock protein involved: (**A**) *Candida albicans* (Hsp110, Hsp90, and Hsp70); (**B**) *Cryptococcus neoformans* (Hsp104, Hsp90, and Hsp70); (**C**) *Sporothrix schenckii* (Hsp90, Hsp70, and Hsp60); (**D**) *Paracoccidioides brasiliensis* (Hsp90, Hsp60, and Hsp40); (**E**) *Aspergillus fumigatus* (Hsp90 and Hsp70); (**F**) *Histoplasma capsulatum* (Hsp70 and Hsp60); (**G**) *Lomentospora prolificans* (Hsp90 and Hsp70); (**H**) *Trichosporon asahii* (Hsp90); (**I**) *Rhizopus arrhizus* (Hsp90); (**J**) *Mucor circinelloides* (Hsp90); (**K**) *Coccidioides immitis* (Hsp60); (**L**) *Talaromyces marneffei* (Hsp90); (**M**) *Trichophyton rubrum* (Hsp90); (**N**) *Malassezia sympodialis* (Hsp105). Functions marked with an asterisk (*) correspond to proposed or hypothesized roles for which there is not yet sufficient experimental evidence.

During the infective process of pathogenic fungi, they face diverse types of stress imposed by the host, including physical barriers, such as epithelia, the secretion of antimicrobial peptides, the action of immune effectors, and thermal stress. However, pathogenic fungi have evolved strategies to cope with these stress inducers, adapting and surviving within the host (Figure 1).

### 4.1. Hsp110

Although it has not yet been elucidated whether Hsp110 plays a role in the virulence of pathogens such as *C. albicans* and *C. neoformans*, these proteins have an indispensable role in fungal biology through their “holdase” activity. This function prevents the formation of denatured protein aggregates and facilitates their refolding to return to their native form, maintaining cellular proteostasis [52].

In *C. albicans*, one Hsp110 has been reported, identified as Msi3, and research has focused on generating conditional mutants, since it is a single-copy and essential gene in this species [53]. Biochemical studies have shown that Msi3 functions as an NEF, preventing protein aggregation and assisting in protein refolding under stress conditions. These studies have linked this chaperone with cell growth through the calcineurin signaling pathways [36,54]. In *C. neoformans*, although an Hsp110 homolog has not been specifically characterized, Hsp104 was immediately overexpressed under thermal stress conditions above 55 °C, forming part of the regulatory network that enables the fungus to survive extreme temperatures. Hsp104 also contributes to adaptation under stress and has been linked to virulence-related processes in other pathogenic fungi, such as biofilm formation and survival in host environments. Although it is discussed in this section for its involvement in the heat stress response in *C. neoformans*, Hsp104 belongs phylogenetically and structurally to the Hsp100/Clp ATPases family [55]. The recombinant version of *M. sympodialis* Hsp105 shows a 69% binding frequency to human IgE present in the sera of AEDS patients. This result confirms Hsp105 as an antigen capable of triggering an allergenic immune response [32].

Msi3 is also related to processes of resistance to fluconazole. Within the calcineurin signaling pathways, fluconazole induces the accumulation of intracellular Ca^2+^, which positively induces Msi3 expression and modulates the expression of UTR2 and PLC3, components of the calcineurin signal transduction pathway that activate genes involved in resistance to azole antifungals [54]. Msi3p acts as a co-chaperone through NEF for other chaperones (Hsp70 and Hsp90), which have also been linked to fluconazole resistance through the calcineurin pathway (Table 1) [54].

The functions of the Hsp110 family vary among fungal species but converge on stress adaptation and cellular proteostasis. In *C. albicans*, participates in calcineurin signaling pathways associated with cell growth and fluconazole resistance. In *C. neoformans*, Hsp104 is strongly induced under extreme thermal stress and contributes to fungal adaptation and virulence-related processes. In *M. sympodialis*, Hsp105 has been identified as an immunologically relevant antigen. Together, these observations illustrate how the Hsp110 family displays functional diversity in virulence but maintains a central role in stress adaptation and fungal survival.

### 4.2. Hsp90

Hsp90 is a molecular chaperone that, in its intracellular form, is involved in the correct folding of nascent ribosomal proteins, maintaining protein homeostasis under stress conditions, and regulating and stabilizing different signaling transducers that control gene expression of different virulence factors [56]. In *C. albicans* and *C. auris*, Hsp90 has been reported as part of the mechanism of stress response and adaptation, including regulation of cell signaling processes in morphogenesis [57,58]. In *C. albicans*, Hsp90 controls the machinery for the transition to filamentous growth by modulating signaling cascades such as the Ras1-cAMP-PKA pathway and interacting with cell wall integrity pathways, which are crucial virulence traits for tissue invasion and immune evasion [57]. In *S. schenckii* and *P. brasiliensis*, Hsp90 has only been indirectly related to the regulation of thermal and oxidative stress associated with the dimorphic transition from saprophytic mycelium to infective yeast. This dimorphism is mediated by Hsp90 and the calcium/calmodulin kinase 1 signaling pathway [59,60,61]. The Hsp90 role in *A. fumigatus* is required in morphological processes important for tissue infection, including conidiation, hyphal growth, and germination. Hsp90 also contributes to cell wall synthesis and stability, mainly in β-1,3-glucan synthesis, and regulates the transcription factors brlA, abaA, and wetA involved in conidiation and melanin pigmentation. Hsp90 mutants show defects in the formation of melanized conidia, hyphal growth, and germination [62]. In *Cryptococcus*, pharmacological inhibition or genetic depletion of Hsp90 impairs growth at host temperature, compromises cell wall integrity, affects capsule assembly and maintenance, and inhibits biofilm formation. This was explained because in the Hsp90 depletion, there was less cell growth due to defects in adequate development at 37 °C, not only affecting the generation of new cells but also having clear defects in the formation of the glucuronoxylomannan capsule (Table 1) [56,63,64].

Fungal Hsp90 is implicated in antifungal drug resistance. In *C. albicans*, *C. neoformans*, and *A. fumigatus*, Hsp90 regulates resistance through stabilization of signaling pathways, such as calcineurin and the protein phosphatase signaling pathway and the mitogen-activated protein kinase Mkc1 pathway (Pck1-MAPK), in which the transcription factor Crz1 is activated to confer echinocandin resistance [54,56,63,65,66]. Additionally, the calcineurin pathway activates an antifungal resistance system in *A. fumigatus* biofilms, promoting extracellular matrix accumulation and DNA release [67]. Hsp90 localization in the *C. neoformans* cell wall is related to the natural resistance to echinocandins, and its pharmacological inhibition/depletion increased the sensitivity to fluconazole. Similarly, Hsp90 inhibition in *C. auris* and *C. albicans* converts fluconazole from fungistatic to fungicidal, reducing growth and virulence in a murine model of candidiasis (Table 1) [56,63,66,68]. Hsp90 is a part of the stress response machinery in many fungi. In *T. rubrum*, it regulates the expression of virulence factors, such as PacC, which controls pH adaptation and keratin degradation. At the same time, Hsp90 regulates the response to antifungal drugs. Therefore, inhibition of Hsp90 in *T. rubrum* by chemical compounds increases susceptibility to antimycotic drugs, like itraconazole and micafungin, in the treatment of nail mycosis in vitro [30,31]. In the case of *L. prolificans* and *R. arrhizum*, these fungi exhibit inherent resistance to antifungal drugs, including triazoles. This resistance is mediated by the calcineurin pathway, where the inhibition of Hsp90 generates drug hypersensitivity and induces apoptosis in the cell [19,21]. Overall, Hsp90 plays a key role in promoting antifungal resistance in many fungi with different lifestyles.

Hsp90 is not only related to signaling processes and stress responses, but it also plays an important role as an immunomodulator. In *C. neoformans*, it has been identified that Hsp90, as an antigen of the humoral response in pulmonary cryptococcosis, determines the capsule size [57,64]. Hsp90 has been detected in the *C. albicans*, *A. fumigatus*, and *P. brasiliensis* cell walls and carries out secondary functions, such as cell wall stabilization. In *T. marneffei*, Hsp90 participates in the process of DOPA-melanin synthesis, where this chaperone is upregulated in melanized yeast cells [24]. In mucoralean fungi *R. arrhizum* and *M. circinelloides*, the calcineurin/Hsp90 pathway has been suggested to be related to a transient hypervirulence state in response to mechanical stress [21]. In *C. albicans*, Hsp90 is in the extracellular medium, where processing occurs at Leucine 312, generating a fragment of 47 kDa from the CTD region [57]. This fragment is a primary antigenic determinant that generates a protective immunity against candidiasis in mice immunized with peptides derived from this Hsp90 fragment [69]. During the infectious process, a greater release and processing of Hsp90 occurs when cells interact with macrophages, due to *C. albicans* lysis, activating the NF-κB signaling pathway, which occurs through the detection of Hsp90 by TLRs on the macrophage surface. This leads to NLRP3 inflammasome activation, which can subsequently induce caspase-1 activity and promote macrophage pyroptosis [57,70]. In the case of *P. brasiliensis*, Hsp90 was recently identified on the cell surface, having a possible function as an immunomodulator, since the use of specific monoclonal antibodies against Hsp90 in an in vivo murine model showed a protective effect against the invasion of this fungus (Table 1) [71].

In *C. albicans*, *C. neoformans*, and *A. fumigatus*, Hsp90 mainly regulates stress response, morphogenesis, and antifungal drug resistance through signaling pathways like calcineurin and MAPK. In contrast, in dimorphic fungi, such as *S. schenckii* and *P. brasiliensis*, Hsp90 is more related to the regulation of thermal and oxidative stress associated with the dimorphic transition from mycelium to yeast. In other fungi, like *T. marneffei* or mucoralean species, Hsp90 participates in more specific processes, including melanin synthesis or transient hypervirulence states. These observations suggest that, although the stress response function of Hsp90 is conserved, its role in virulence and host adaptation can vary between fungal species [71].

### 4.3. Hsp70

Many proteins are classified as moonlighting proteins, which can develop another biological function, different from their canonical one, carried out in the same location or another part of the cell. In many pathogenic fungi, different moonlighting proteins have been identified as adhesins on the surface that mediate the interaction with the host. Examples include Pap1 and Gp70 in *S. schenckii* [72,73,74,75], and Scf1 and Gpm1 in *Candida* spp. [76,77]. Chaperones have also been identified as surface proteins with the ability to adhere to components of the host’s extracellular matrix. *C. albicans* Hsp70 (Ssa1) can bind to the E-cadherin of epithelial cells and the N-cadherin of endothelial cells, mediating *C. albicans* endocytosis and infection. Ssa1 and other Hsp70 family members act as invasins by interacting directly with host cadherins and triggering host cell cytoskeletal rearrangements required for fungal internalization. Null mutants and specific anti-Hsp70 antibodies significantly reduced adhesion to epithelial and endothelial cells in vivo, attenuating virulence in murine infection models [78,79,80]. In *C. neoformans*, Hsp70 localization in the cell wall, under thermal stress, is related to thermotolerance and capsule development, both necessary for host invasion and survival under hostile host conditions [56]. Hsp70 is also secreted and may interact with host receptors, including components of the TLR4/CD14/MD2 complex, facilitating adhesion to the alveolar epithelial cells, which also have an affinity toward the *C. neoformans* capsule [81]. In a study of differential gene expression under metal-induced oxidative stress in the opportunistic fungus *T. asahii*, Hsp70 was found to be upregulated in response to sodium arsenite (NaAsO_2_). This chaperone is part of the mechanism involved in pathogen adaptation to oxidative stress, such as that imposed by arsenite exposure or by the host environment [20].

In *A. fumigatus*, the Hsp70-StiA-Hsp90 complex is essential for tolerance to the echinocandin caspofungin, mediated through the calcineurin signaling pathway and cell wall integrity responses [82]. In strains of *Aspergillus terreus* and *A. fumigatus* resistant to amphotericin B, Hsp70 is overexpressed in the drug’s presence and the inhibition of this chaperone reduces the minimum inhibitory concentration of amphotericin B and improves *Galleria mellonella* survival, demonstrating the relevance of Hsp70 in antifungal resistance mechanisms [83]. A similar response occurs in *L. prolificans*, where Hsp70 is upregulated under conditions of drug stress induced by treatment with voriconazole. This response may be regulated by the calcineurin pathway as part of the cellular response to cell wall stress [19,84]. In *S. schenckii*, Hsp70 is part of oxidative stress response mechanisms [85]. In cell wall structural studies, Hsp70/DnaK has been found extracellularly, as part of the cell wall peptidorhamnomannan fraction; although its precise function is unknown, it is speculated that it could be acting as an adhesin and immunomodulator, as is already known for other proteins of this chaperone family (Table 1) [73].

The surface Hsp70 (Ssa1) in *C. albicans*, *C. neoformans*, and *H. capsulatum* not only plays a role as a virulence factor relevant for establishing infection but also participates in immunomodulation and evasion of the host innate immune system. Hsp70 can generate an anti-inflammatory environment, mainly by inducing dendritic cells to favor anti-inflammatory cytokine profiles, reducing IL-12 and IL-4, and limiting the recruitment of other immune cells. It can also interact with co-stimulatory molecules CD80 and CD86 and the CTLA-4 receptor on T cells, contributing to an inhibitory state of the adptative immune response [86,87]. In *C. neoformans*, Hsp70 participates in immune evasion strategies by polarizing macrophages toward an M2 (anti-inflammatory) phenotype, promoting evasion of other innate defense systems, which allows it to proliferate in the alveoli and subsequently migrate to the brain through the circulatory system. *HSP70* deletion has been found to decrease the fungal load in the lungs and the correct activation of alveolar M1 macrophages (proinflammatory) [17,46]. Hsp70 is also a predominant antigen on the surface of *C. neoformans* yeast cells, activating macrophage responses via TLR2 and TLR4 engagement [81,88]. Similarly, in *C. albicans*, secretion of Hsp70 under hypoxic conditions facilitates interaction with innate immune cells through TLR2 and TLR4, and cytokine production, inducing the anti-inflammatory IL-10 and the pro-inflammatory IL-6. The latter, although it induces an inflammatory effect by regulating the transcription factor NF-κB, inhibits the exacerbated antifungal response by M0 and M1 macrophages [87]. The *H. capsulatum* surface Hsp70 can activate the immune system, in particular the proliferation of T cells, but its high identity percentage (71%) with the human ortholog means it does not have the capacity to generate protective immunity when placed as a prophylactic agent in an in vivo model (Table 1) [89].

There is a peculiar group of HSPs (80–87 kDa) that are structurally similar to Hsp70, but they have not been classified as a group of chaperones. Proteins of 80 kDa and 87 kDa have been identified in the species *H. capsulatum* and *P. brasiliensis*, respectively. These are relevant proteins for the interaction with the host, since the development of a monoclonal antibody showed that these proteins are constitutively expressed, but increase in abundance when the fungi are under conditions of thermal stress (Table 1) [90,91].

In pathogenic fungi, such as *C. albicans*, *C. neoformans*, and *H. capsulatum*, surface Hsp70 acts mainly as an adhesin and immunomodulator, mediating interaction with host receptors and contributing to immune evasion. In contrast, in filamentous fungi such as *A. fumigatus* and *L. prolificans*, Hsp70 is more closely associated with tolerance to antifungal drugs and cell wall stress response mediated by calcineurin signaling. In other species, such as *T. asahii* and *S. schenckii*, Hsp70 appears to play an important role in adaptation to environmental stress conditions, including oxidative stress. In conclusion, Hsp70 functions are conserved in stress response and the contribution to virulence, drug resistance, and host interactions depends on the species.

### 4.4. Hsp60

Like other chaperones, Hsp60 is found on the cell surface with functions beyond its canonical role in mitochondrial protein folding and homeostasis. One of the functions attributed to the surface Hsp60 in *H. capsulatum*, *C. immitis*, and *S. schenckii* is its role as an adhesin to different host components [23,73,92]. In the case of *S. schenckii*, recombinant Hsp60 was analyzed for its interaction with the different components of the extracellular matrix, showing binding to different ligands, such as fibrinogen, laminin, elastin, and fibronectin. These ligands are structural proteins of host tissues, suggesting that Hsp60 contributes to fungal adhesion and colonization during early infection. The ability to bind multiple ECM components indicates that Hsp60 may function as a multifunctional adhesin facilitating tissue invasion and dissemination. Furthermore, in a *G. mellonella* infection model, the administration of this protein, before fungal inoculation, induced an immunological priming and improved infection control of *S. schenckii* infection [73].

*H. capsulatum* has been identified as the ligand mediating binding to the CD18 receptor (part of the CD11b/CD18 integrin complex, also known as CR3) on human macrophages [92]. This interaction promotes fungal uptake by macrophages; however, rather than leading to efficient killing, it facilitates intracellular survival, which is common in *H. capsulatum* pathogenesis. The binding of Hsp60 to CD11b/CD18 promotes cellular responses in macrophages and contributes to the modulation of cytokine production. The use of recombinant Hsp60 as a vaccine demonstrated protective immunity in murine models, mediated by CD4+ and CD8+ T lymphocytes and associated with the expression of the cytokines IL-12, IL-10, and IFN-γ [18,92,93]. IL-12 and IFN-γ are indispensable for the polarization and activation of Th1 macrophages, promoting fungal clearance, whereas IL-10 may regulate excessive inflammation. A similar phenomenon occurs with Hsp60 of *C. immitis*. This chaperone shows 78% similarity to Hsp60 of *H. capsulatum*. Recombinant Hsp60 from *C. immitis* induces the production of cytokines associated with a Th2 immune profile (IL-4, IL-5, and IL-10). This immune response promotes the recruitment of B cells and the development of adaptive immunity, while IL-10 can suppress the Th1-mediated response. These findings indicate that Hsp60 plays a dual role as both a virulence factor involved in macrophage interaction and a potent immunogenic molecule capable of inducing protective cellular immunity [18,92,93]. The importance of Hsp60 in *P. brasiliensis* has also been described in relation to morphological transition. *HSP60* gene expression is significantly higher in the yeast phase than in mycelia phase, suggesting a role in adaptation to host temperature and in maintaining the pathogenic yeast morphology [94]. The use of the DNA-Hsp65 vaccine derived from *Mycobacterium tuberculosis* has been evaluated against infections caused by *P. brasiliensis* and *F. pedrosoi* in murine models. This vaccination stimulates a Th1 response, with the increased production of IL-12 and INF-γ, as well as the production of nitric oxide (NO), an antimicrobial metabolite (Table 1) [22,29].

The particular functions of Hsp60 in species such as *H. capsulatum*, *C. immitis*, and *S. schenckii,* acting as an adhesin that mediates interaction with host components and facilitates invasion, depend on the fungal species. In *H. capsulatum,* Hsp60 interacts with the CD11b/CD18 receptor in macrophages, promoting fungal internalization and inducing a protective Th1 immune response. In contrast, the recombinant Hsp60 from *C. immitis* induces a Th2 cytokine profile. In conclusion, the role of Hsp60 in the virulence of fungi is mainly in adhesion and immunomodulation response.

### 4.5. Hsp40

The chaperone Hsp40 was originally identified in *S. cerevisiae* as the protein Ydj1p, a member of the *HSP40/DnaJ* family. Together with other genes, such as *MDJ1*, *SCJ1*, *SIS1*, and *SEC63*, it composes the *HSP40* gene family in yeast. The proteins have different subcellular localization, performing their function as a co-chaperone of Hsp70, simulating its ATPase activity and facilitating substrate binding [95,96]. Ydjp1 is particularly important for thermotolerance, as demonstrated by the requirement of its farnesylation for proper function at elevated growth temperatures. This highlights the central role of Hsp40 proteins in stress adaptation and protein quality control under heat shock conditions. In *P. brasiliensis,* Mdj1/Hsp40 is located not only in mitochondria but also in the cell wall, tightly bound to it. The function it performs in this extracellular location is not clear yet; however, its presence in the cell wall allows us to hypothesize about a possible moonlighting activity, being potentially involved in fungal virulence, host interaction, adhesion, immune modulation, or environmental stress sensing, as is the case with other HSPs (Table 1) [96].

**Table 1:** Role of heat shock proteins in fungal virulence.

HSP	Species	Function/Role	Citation
Hsp110 (Msi3p)	*Candida albicans*	Holdase activity (prevents protein aggregates), co-chaperone of Hsp70/Hsp90, cell growth regulation, fluconazole resistance via calcineurin pathway	[36,52,53,54]
Hsp105	*Malassezia sympodialis*	RHsp105 binding to human IgE in sera of AEDS patients	[32]
Hsp104 (Hsp100/Clp)	*Cryptococcus neoformans*	Overexpression under thermal stress >55 °C, part of the regulatory network for survival at high temperatures	[55]
Hsp90	*C. albicans*, *Candida auris*	Regulation of morphogenesis, stress adaptation, antifungal resistance, immunomodulation, biofilm formation	[57,58,66,68,69,70]
	*Aspergillus fumigatus*	Morphogenesis, cell wall development and stability, β-1,3-glucan formation, melanization, conidiation	[62]
	*C. neoformans*	GXM capsule formation, biofilm development, virulence, immunomodulation	[56,63,64]
	*Paracoccidioides brasiliensis*	Immunomodulation, protective effect via monoclonal antibodies	[71]
	*Rhizopus arrhizus*	Triazole drug resistance via the calcineurin pathway, transition to hypervirulent state in response to mechanical stress	[21]
	*Mucor circinelloides*	Transition to hypervirulent state in response to mechanical stress	[21]
	*Lamentospora prolificans*	Triazol drug resistance via the calcineurin pathway	[19,21]
	*Talaromyces marneffei*	Synthesis of DOPA-melanin in yeast cells	[24]
	*Trichophyton rubrum*	Regulates expression of PacC virulence factor, pH adaptation, keratin degradation, and resistance to itraconazole and micafungin	[30,31]
Hsp70 (Ssa1)	*C. albicans*	Adhesion to epithelial/endothelial cells, mediates endocytosis, immune evasion	[78,79,80,87]
	*C. neoformans*	Thermotolerance, GXM capsule development, adhesion to epithelial cells	[56,81,88]
	*A. fumigatus*	Hsp70-StiA-Hsp90 complex, caspofungin resistance via calcineurin pathway, maintains cell wall integrity	[82]
	*Aspergillus terreus*	Overexpression under amphotericin B, antifungal resistance	[83]
	*Sporothrix schenckii*	Oxidative stress response, potential adhesin and immunomodulator	[73,85]
	*Histoplasma capsulatum*	Immunomodulation, interaction with the host immune system, T cell proliferation	[86,89]
	*L. prolificans*	Upregulated in response to drug resistance to Voriconazole	[19,84]
	*Trichosporon asahii*	Upregulated in adaptation to oxidative stress	[20]
Hsp80/Hsp87	*H. capsulatum/P. brasiliensis*	Host interaction; upregulated under thermal stress	[90,91]
Hsp60	*H. capsulatum*	Adhesin, immunomodulation, protective immunity in murine models, induces IL-12, IFN-γ, and IL-10	[18,92,93]
	*S. schenckii*	RHsp60 multispecific adhesin (fibrinogen, laminin, elastin, fibronectin), protection in the *Galleria mellonella* model	[73]
	*Coccidioides immitis*	Adhesion to host components, induces Th2 immune profile (IL-4, IL-5, and IL-10)	[18,92,93]
Hsp40 (Ydj1p/Mdj1)	*Saccharomyces cerevisiae/P. brasiliensis*	Hsp70 co-chaperone; potential moonlighting function in cell wall, virulence, or host interaction	[95,96]

## 5. Drugs with the Ability to Inhibit HSPs

Antifungal treatment is mainly carried out with the use of three classes of drugs: polyenes (amphotericin B), which target plasma membrane ergosterol, generating pores; azoles, which inhibit the enzymes of ergosterol synthesis; and echinocandins, which inhibit cell wall β-1,3-glucan synthesis. The increase in resistance to these antifungal drugs is due to increased activity of ergosterol synthesis enzymes, increased expression of efflux pumps, and overexpression of biosynthetic enzymes of structural components, among others [58,97]. This represents a problem for public health; therefore, possible therapeutic targets have been sought for the treatment of these fungal infections, including heat shock proteins. Although they are highly conserved in all organisms, some compounds have been developed that inhibit the function of these chaperones and, therefore, allow the control of the growth, spread, and resistance of these fungi (Table 2) [65].

### 5.1. Targeting the Hsp90 NTD

The Hsp90 NTD is the main catalytic site, where the ATP-binding pocket is located to perform the ATPase function, so inhibition of this region provides an opportunity for the treatment of fungal infections. All drugs within this group are synthetic and function competitively with the ATP-binding site on the Hsp90 NTD, directly inhibiting their catalytic activity. These include drugs based on resorcinol, purine scaffolds, some substituted benzamides, and pyrimidine derivatives [39]. The most notable of these inhibitors is radicicol (Table 2) [37].

Geldanamycin

Geldanamycin is a benzoquinone-type ansamycin drug, which has been widely investigated in cancer therapy for its function as an Hsp90 inhibitor in cancer cells. It works by directly binding to the ATP-binding pocket in the NTD domain that these chaperones have and inhibiting their functions within the signaling pathways in response to stress, due to the high homology and conservation of these chaperones in all kingdoms of life. In fungi, such as *C. albicans*, *A. fumigatus*, and *S. schenckii*, it has been used at concentrations of 1 to 10 µM, inhibiting the signaling cascade of MAPK kinases and, in turn, inhibiting dimorphism at high temperatures [39,62]. This drug blocks the function of Hsp90, inducing the inhibition of the calcineurin pathway in *L. prolificans* and *R. arrhizus*, in response to azole antifungal drugs, which leads to the activation of apoptotic pathways due to the accumulation of ROS in the cell [19]. In *T. marneffei*, it inhibits the correct synthesis of DOPA-melanin, generating yeast cells that lack the characteristic brown-black pigment [21,24]. It has also been analyzed in synergy with different antifungal drugs, determining that by inhibiting the function of Hsp90 in these fungi, the generation of resistance to azole and echinocandin-type antifungals was restricted, with negative consequences for the dissemination of the infection in a mouse model. There are certain derivatives of this, which include 17-AAG (17-(allylamino)-17-demethoxygeldanamycin), 17-DMGA (17-dimethylaminoethylamino-17-demethoxygeldanamycin), IPI-493, and IPI-504 (Table 2) [39,62].

2′,4′-Dihydroxychalcone

It is an inhibitor with the ability to bind to the NTD domain, specifically to the Hsp90 ATP-binding pocket. In *A. fumigatus*, it showed moderate fungicidal activity, affecting mycelial growth and biofilm formation, as well as a defect in the cell wall β-1,3-glucan content. This effect is related to the role that Hsp90 plays in the calcineurin pathway, which is responsible for maintaining cell wall integrity during interaction with echinocandins. In addition, it has a direct effect on the conidial melanization process, which is an important virulence factor to tolerate extreme conditions. The effect of this inhibitor increased in synergy with commonly used antifungal drugs, like itraconazole and caspofungin, due to a dual effect, inhibiting the calcineurin pathway and affecting cell wall and plasma membrane biosynthetic enzymes (Table 2) [98].

5′-N-Ethylcarboxamidoadenosine

It is an inhibitor that has been tested on different types of 90 kDa chaperones present in different organisms, namely, Grp94, Hsp90α in mammals, Hsp82 in yeast, and HtpG in bacteria. Like many other inhibitors of this particular chaperone, it binds primarily to NTD, specifically to the conserved ATP-binding pocket, thereby inhibiting its ATPase activity and interfering with the conformational cycle required for client protein maturation (Table 2) [99].

Artesunate (ART)

This is a semisynthetic compound derived from the sesquiterpene lactone artemisinin. It was originally developed as an antimalarial drug; however, due to the increase in antifungal resistance in some fungi, a repurposing strategy for this drug has been put in place. Recent studies have reported its antifungal activity by Hsp90 and the calcineurin pathway inhibition, influencing fungal growth, cell wall, and plasma membrane integrity. This led to direct effects on cell adhesion to the host, the development of biofilms, and resistance to antifungal drugs. In synergy with conventional antifungal drugs, such as amphotericin B, ART shows a more visible effect against *A. fumigatus*, *C. neoformans*, and *C. gatti* [99]. In addition, ART has been associated with modulation of the exacerbated inflammatory response caused by some fungal pathogens, particularly *A. fumigatus*. It has been shown to inhibit the production of pro-inflammatory cytokines, including IL-1β, TNF-α, and IL-6, thereby reducing tissue inflammation. In a keratitis model, ART improved disease outcome by inhibiting fungal activity and activating the autophagy pathway, which contributes to the reduction in corneal inflammation (Table 2) [99].

Ganetespib

Ganetespib is an Hsp90 inhibitor, which has been tested in synergy with antifungal drugs, showing inhibition of *C. albicans* growth in combination with fluconazole at a concentration of 8 µg/mL. However, it did not show the same effect on other *Candida* species, such as *C. auris* and *C. glabrata*, in which there was no significant growth inhibition. In *C. albicans*, it inhibited biofilm formation, not through an effect on morphological differentiation to hyphae, but rather by modulating the expression of genes encoding adhesins required for biofilm development [58]. This suggests that antibiofilm activity is linked to transcriptional regulation of adhesin-related pathways rather than to interference with filamentation. This inhibitor acts directly on the ATP-binding pocket located in the NTD of Hsp90, a region highly conserved among Hsp90 homologs in different organisms (Table 2) [58].

Luteolin and Rutin

Some molecular analyses demonstrated that compounds deviated from *Cardiospermum halicacabum* show affinity for the ATP-binding site of the Hsp90. These compounds have also shown growth inhibition in bacteria. The analysis resulted in the identification of 13 compounds, of which only 2 have potential as inhibitors of Hsp90 in *T. rubrum*. Luteolin-7-O-glucoside and rutin exhibited antifungal activity at concentrations of 250 and 500 µg, respectively, comparable to the antifungal activity of amphotericin B at concentrations of 1.5 and 3 µg [30].

### 5.2. Targeting the CTD

The Hsp90 CTD does not have highly conserved sequences between phylogenetically distant organisms, so the development of specific inhibitors toward this domain would expand the current antifungal drug repertoire. Some of these inhibitors are mainly derived from natural compounds [39]. It should be noted that most of these have only been tested in five *Candida* species (*C. albicans*, *C. glabrata*, *C. tropicalis*, *C. parapsilosis*, and *Candida krusei*). Therefore, our knowledge of them is restricted to these species, and their function in filamentous fungi is not yet clear. This type of compound offers an advantage over conventional antifungal drugs, since they do not present immediate resistance, so they can be used synergistically or independently (Table 2) [100].

Green tea catechin

This compound has the capacity to inhibit the growth of species such as *C. albicans*, *C. glabrata*, *C. tropicalis*, and *C. krusei*. It showed a greater effect on *C. glabrata* and *C. krusei*, with inhibitory concentration 50 (IC50) values ranging from 0.82 to 5.3 µM. In contrast, for *C. albicans* and *C. tropicalis*, the IC50 values were in higher ranges, from 15.1 to 58.1 µM. For *C. parapsilosis*, no growth inhibition was shown. These data reveal that these types of inhibitors are species-dependent, by mechanisms underlying the internalization of the compound or by its own affinity to the target protein (Table 2) [39].

[3,4-b]Pyridine (2H)

The 2H, [3,4-b]Pyridine derivative of pyrazol has proven to be an effective inhibitor of Hsp110 in *C. albicans*, *C. auris*, and *C. neoformans*, acting by binding to the SBD of Hsp110, reducing its affinity to the protein client. This interaction inhibits the holdase function of tHsp110, which is essential for preventing aggregation of unfolded or misfolded proteins under stress conditions. Consequently, denaturation accumulated in the cytosol, leading to proteotoxic stress and cytotoxicity. Therefore, fungal cell growth and viability are significantly affected (Table 2) [52,100].

**Table 2:** Heat shock protein inhibitors with antifungal activity.

Inhibitor	Fungal Species Tested	Observations	Citation
Targeting the Hsp90 N-terminal domain			
Geldanamycin	*Candida albicans*	Inhibits Hsp90 function; synergizes with azoles and echinocandins; reduces antifungal resistance	[39]
	*Aspergillus fumigatus*	Inhibits Hsp90-dependent signaling and stress adaptation; synergizes with antifungal drugs	[62]
	*Sporothrix schenckii*	Inhibits dimorphic transition at elevated temperature	[39]
	*Lamentospora prolificans*	Inhibits calcineurin/Hsp90 pathway; increases azole susceptibility and induces ROS-associated apoptosis	[19]
	*Rizopus arrhizus*	Inhibits calcineurin/Hsp90 pathway; promotes drug hypersensitivity and apoptosis	[19,21]
	*Talaromyces marneffei*	Impairs DOPA-melanin synthesis and melanization	[24]
17-AAG/17-DMGA/IPI-493/IPI-504	—	Similar mechanism to Geldanamycin; developed for improved solubility and reduced toxicity	[39,62]
(Geldanamycin derivatives)			
2′,4′-Dihydroxychalcone (2′,4′-DHC)	*A. fumigatus*	Moderate fungicidal activity; inhibits biofilm formation and mycelial growth; affects β-1,3-glucan synthesis and melanin production; synergistic with itraconazole and caspofungin	[98]
5′-N-Ethylcarboxamidoadenosine	Yeast (*Hsp82*);	Inhibits ATPase activity; cross-species inhibition of Hsp90 family members	[99]
	bacteria (*HtpG*); mammals (*Hsp90α*)		
Artesunate (ART)	*A. fumigatus*	Antifungal and anti-inflammatory activity; disrupts cell wall and plasma membrane; reduces adhesion and biofilm formation; synergistic with amphotericin B; decreases IL-1β, TNF-α, and IL-6	[99]
	*Cryptococcus neoformans*		
	*Cryptococcus gattii*		
Ganetespib	*C. albicans*	Inhibits *C. albicans* growth synergistically with fluconazole; blocks biofilm formation via adhesin gene suppression; no effect on *C. auris* or *C. glabrata*	[58]
	*Candida auris*		
	*Candida glabrata*		
Luteolin and Rutin	*Trichophyton rubrum*	Antifungal activity at concentrations of 250 and 500 µg, comparable to the activity of amphotericin B at 1.5 and 3 µg	[30]
Targeting the Hsp90 C-terminal domain			
Green tea catechin	*C. albicans*	Inhibits growth in a species-dependent manner; strongest inhibition in *C. glabrata* and *C. krusei* (IC_50_ = 0.82–5.3 µM); weaker in *C. albicans* and *C. tropicalis*	[39]
	*Candida glabrata*		
	*Candida tropicalis*		
	*Candida krusei*		
[3,4-b]Pyridine (2H)	*C. albicans*	Reduces substrate affinity; inhibits holdase function; causes protein aggregation and cytotoxicity under thermal stress; fungicidal effect	[52,100]
	*C. auris*		
	*C. neoformans*		

## 6. Conclusions

Heat shock proteins have proven to be essential molecules in the biology of mammalian fungal pathogens, playing a crucial role in adapting to various types of host-induced stresses and contributing to their pathogenic success. Besides their canonical functions, HSPs participate in morphogenetic transition, cell wall maintenance, biofilm development, resistance to various antifungal drugs, and modulation of the host’s immune response.

Among the evidence found, Hsp90 stands out due to its extensive involvement as a regulator of a wide range of target proteins, some essential signaling components in different stress responses, as well as in the fungus’s pathogenic characteristics. Similarly, chaperones, such as Hsp70 and Hsp60, participate not only in proteostasis but also as key intracellular and surface components for host interaction, including cell adhesion and immune modulation. Conversely, Hsp110 and Hsp40 remain less explored as virulence-associated proteins, with research focusing primarily on their role in proteostasis. This leaves gaps in our understanding of their potential involvement in these processes.

The global increase in invasive fungal infections and the growing emergence of resistance to known antifungal drugs underscore the urgent need for alternative therapeutic strategies. In this context, pharmacological inhibition of fungal HSPs has proven to be a promising approach. Pharmacological Hsp90 inhibition increases susceptibility to stress by altering the various stress responses, sensitizing fungi to existing antifungal drugs, and reducing pathogenic traits in multiple models. The synergistic use of these inhibitors with currently available antifungal drugs offers a viable alternative to curb the rise in infection cases and resistance acquisition.

Future studies on HSP species-specific functions, surface-associated activities, and host-pathogen interactions will be essential to fully understand their potential as therapeutic targets and develop more effective antifungal interventions.

## Data Availability

Data are contained within the article.
